# Value of Contrast-Enhanced FLAIR Images for the Depiction of Papilledema

**DOI:** 10.5334/jbsr.2479

**Published:** 2021-06-17

**Authors:** Alice Petiot, Thierry Duprez

**Affiliations:** 1 Cliniques Universitaire Saint Luc, BE

**Keywords:** papilledema, intra-cranial hypertension, MRI, FLAIR, contrast-enhanced scanner

## Abstract

**Teaching Point**: Contrast-enhanced FLAIR images have unsurpassed value for the radiological depiction of hypertensive papilledema. FLAIR acquisition should therefore be performed after intravenous contrast, especially in the of work-up of intracranial hypertension and/or tumor.

## Case History

**Figure 1:**
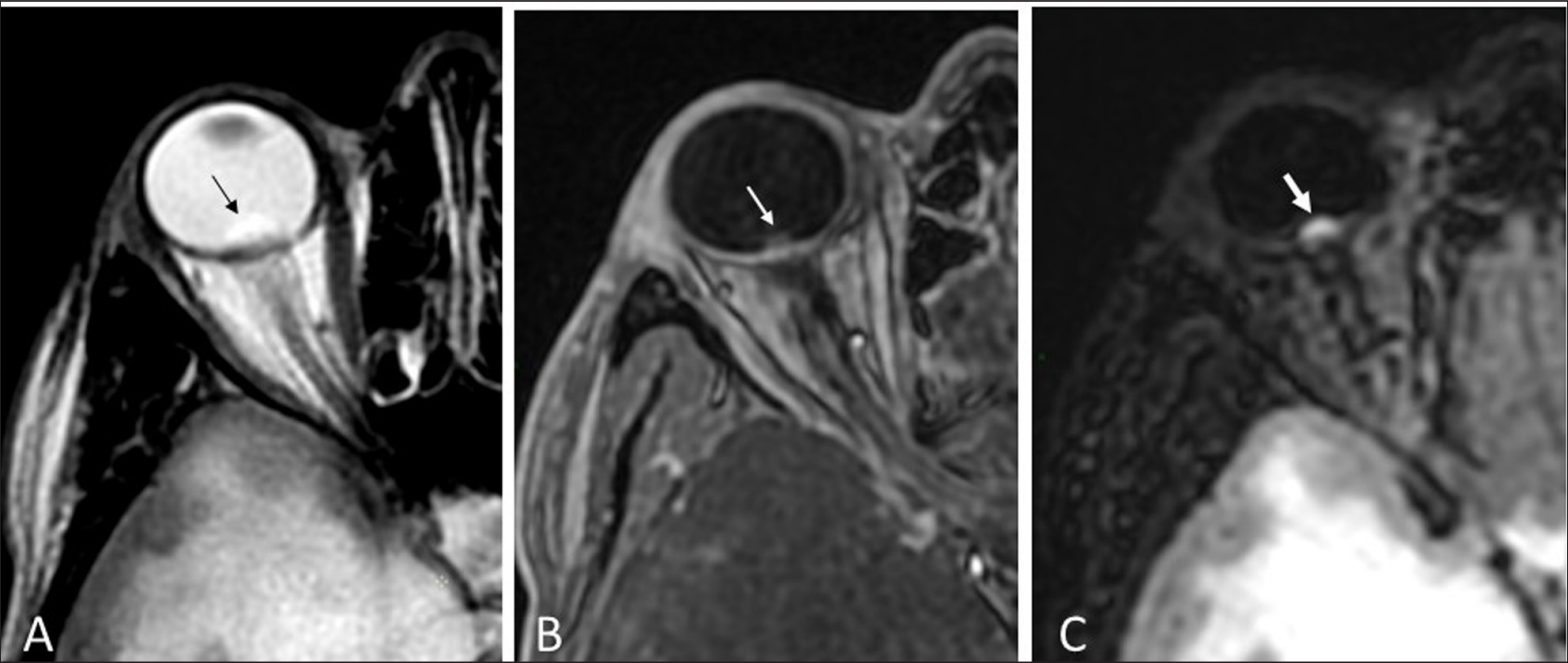


A healthy 31-year-old woman presented with a three-month history of increasing holocranial headaches, weight loss, and nausea. Initial contrast-enhanced (CE) computed tomography (CT) demonstrated a large right-sided temporal malignant tumor surrended by edema. Magnetic resonance (MR) work-up supported the hypothesis of a high-grade glioma (not shown). Edema of the optic disc matching the clinical signs of intracranial hypertension (ICHT) was additionally highlighted on MR. The feature had been suspected on CT (not shown), becoming more obvious on T2-weighted (WI) (***Figure 1A***) and post-contrast T1-WI (***Figure 1B***) images, but far less than on fat-suppressed post-contrast FLAIR (fluid-attenuated inversion recovery) (***Figure 1C***, arrows).

Ophthalmologic examination revealed a bilateral grade IV papilledema and a left lateral homonymous hemianopia. Histopathologic examination of resected specimen revealed a WHO grade II astrocytoma with IDH1 mutation.

## Comment

The exquisite depiction of the papilledema on post-contrast FLAIR views was hypothesized to be synergistically due to both the high protein content and the leakage of contrast agent molecules within the fluid filling the protruding disks. The combined paramagnetic effect of proteins and contrast agent results in a strong signal intensity on FLAIR images, contrasting with nulled signal intensity of the adjacent vitreous fluid. Because of this, contrast-enhanced FLAIR images surpassed all other imaging techniques for the radiological depiction of hypertensive papilledema [1]. FLAIR acquisition should therefore be performed after intravenous contrast, especially in the of work-up of intracranial hypertension and/or tumor.
